# Foreign

**Published:** 1841-07-10

**Authors:** 


### FOREIGN.

The last British and Foreign Medical Review contains an article upon the recent work of Dr. Hodgkin, on the diseases of the mucous membranes, from which we extract the following paragraph. It settles the vexed question of the mode of terminating the bronchi, but it must be borne in mind that it had already been conclusively settled, by the dissection and preparation of Prof. Horner, of the University of Pennsylvania, which prove that each bronchus terminates in a lobule, and that, while the cells of each lobule communicate laterally with great freedom, the cells of different lobules remain distinct.

The third portion of the pulmonary mucous membrane, or that lining the air-cells, will require a more extended notice. The following method, for which the author acknowledges his obligations to the late Dr. Babington, is recommended as well calculated to show the ultimate ramifications and terminations of the bronchial tubes.

"A collapsed portion of a healthy lung should be taken, having as small an incised surface as possible: and, on this account, one of the lobes of the lung of an inferior animal answers remarkably well. This portion of lung should then be injected, from the bronchial tube, with the white of egg, in sufficient quantity to distend it, and render its pleural surface smooth. The bronchial tube and the incised surface are then to be secured by ligature, and the whole boiled for a sufficient length of time firmly to coagulate the albumen. By the same process the cellular membrane is so much softened as greatly to facilitate the separation of the structure of the lung without injuring the albumen, which has taken the impression of the cavities into which it was injected. In this way we may discover that nearly all the bronchial ramifications lose their fine tubular form; when they have arrived at a particular degree of minute subdivision; and that, beyond this point, the injected albumen is infiltrated through a spongy texture, so minute that not only its precise form cannot be made out, but its white colour is lost and converted into a gray, from its intermixture with the structure forming the minute cavities in which it is situated." (p. 79, 80.)

Two opinions have been advanced, as our readers are aware, respecting the terminations of the bronchial tubes; one, that there is a free communication between the air-cells and the cellular tissue of the lungs; the other, that the ultimate ramifications of the bronchi are individually unconnected with each other, or with the surrounding pulmonary texture. It would seem from the above method of examination, as well as from others, as Dr. Hodgkin remarks, that there is a communcation existing among the air-cells, but that this communication is not indefinitely extended throughout the whole pulmonary tissue.

---

*Cases of Empyema.* By Dr. Kilgour.—The following case exhibits in the treatment a circumstance not usual in these cases, though, I think, very necessary in any case under the same state—the giving free exit to air, as well as to purulent matter.

Alexander Clark, æt. 23, a mason, was admitted into St. Luke's ward, Oct. 5th, 1839, complaining of difficulty of breathing and slight pain in his side, attended with great emaciation, much feeling of weakness, want of appetite, looseness of his bowels, and occasional sweatings; skin hot; tongue clean; pulse 104; urine containing an abundant lateritious sediment. Has some cough, and expectorates, without difficulty, a small portion of a clear frothy fluid. Lies almost invariably on his left side, or inclined to it; and the difficulty of breathing is much increased on his attempting to turn to his right side. Respiration 56 in the minute.

The left side of the chest is distinctly fuller and rounder than the right. The *sulci* betwixt the ribs are quite visible on the right side; but the left side presents a smooth uniform fulness. It does not move with respiration, as is the case with the right. No pulsation of the heart can be seen on the left side, and the hand laid on the precordial region can scarcely feel it; but on the right of the sternum, and below the nipple, its pulsations are visible to the eye, and its stroke sharp and strong to the hand.—The integuments, discoloured with blisters which have been applied, are œdematous and pit on pressure. The sound is very dull on percussion over the whole side, and no respiratory murmur can be heard. The left side is fully two inches larger than the other one, the measurement being taken below the nipple. Betwixt the fifth and the seventh ribs, and posterior to their cartilages, there is, to the eye, rather more fulness or elevation of the integuments. The ribs are widely separated, and there is a feeling to the fingers of fluid seated at some depth.

States that he was in ordinary health until four weeks ago, when he came home in the evening from his work in a state of perspiration, and changed his shirt, putting on a cold one, and went out. He came home, and went to bed about his usual hour, and soon after felt a most acute pain in his left side, which continued of the most intense character until six o'clock in the morning, when he sent for a medical gentleman, who immediately bled him largely, which was followed with great relief. He also got large doses of tartar emetic, and on the eighth day after, he was out of bed. On the fourteenth day after the attack he went out, and on returning home, he was seized with cold shivering, which returned upon him more than once. Since this time has felt his breathing short, and the weakness has increased very much. States also, that, a year ago, he coughed up a little blood; but he was never disabled from working.

This was clearly a case of epyema following pleuritus. There was no communication with the lungs, so far as the physical signs or the character of the sputa went. Although not stated in the report, as drawn up by the clerk, yet the right lung was found to be free from disease. On raising him up and bending the body forward, a projection of the integuments betwixt the sixth and seventh ribs became much more distinct, and the feeling of fluctuation very palpable. This, therefore, made the place of the operation, which, after consultation, was resolved on, not that of choice but of necessity; but the situation was sufficiently dependent; and the only further desirable circumstance would have been to have had the presentation of the fluid rather nearer the spine than the sternum. Accordingly, on the third day after an opening was made by my colleague, Mr. Keith, at the projecting part, the integuments, &c. having been divided with a scalpel over the upper edge of the seventh rib, and a canula introduced, through which thirty ounces of thick creamy purulent matter passed in a full stream. The canula was then plugged; but, half an hour afterwards, there not being much relief in the breathing, the canula was withdrawn, and thirty-six ounces more of purulent matter suffered to escape from the wound. It was allowed to run so long as it came in an uninterrupted stream, but when it began to come out in jets it was stopt by laying in a piece of lint, and applying over it slips of adhesive plaster.

October 10th. Passed a quiet night, and slept for several hours; was occasionally troubled with cough; pulse 108. The lint inserted in the wound was taken out, and fifteen ounces of purulent matter escaped; but it was ejected in spirts at each inspiration, and a portion of atmospheric air got entrance; for, on laying the ear to the chest, there is heard a gurgling sound, and at the nipple there is an emphysematous tumour, which rises and falls with respiration. The side is much reduced to the eye.

October 11th. Slept well; pulse 100; lint withdrawn, when twelve ounces purulent matter, somewhat fetid, ran out. Its discharge was assisted by pressing below the diaphragm on that side. The emphysematous swelling is greater around the nipple.

October 12th. The amount of discharge twelve ounces, fetid, but thick.

October 14th. Eight ounces yesterday and the same to day; but the compress came off yesterday, and much fluid escaped in the bed.

October 16th. Was very comfortable yesterday, and the plug was not removed. To-day, on removing the dressing, there has been a discharge of thin fetid purulent matter, to the extent of between two and three pounds. During the night he had great difficulty in breathing, and frequent cough, with abundant expectoration, which he feels coming from the affected side.

On the 17th, the report informs us, that the wound had burst twice since the previous day, and that above a couple of pounds of fluid of the same fetid thin appearance had escaped. On the 20th, he was so much better, that he insisted on getting up to try on his waistcoat, which buttoned over him; a property it did not possess previous to his entering the hospital. No discharge from the wound, which appears to be healed up. On coughing, the integuments around the nipple are much blown out. The side by measurement is only about a quarter of an inch larger than the other. The wound, opened with a probe, gave exit to four ounces of purulent matter. Appetite good; a piece of sponge tent was put into the wound. On the 21st, respiration is not heard on the application of the stethoscope, either anteriorly or laterally. The heart is advancing to the left side, and may be seen in part pulsating to the left of the sternum. Chest sounds very loud on percussion on the left side. The case continued in much the same state; but on the 2d November, the report states, "only a discharge to the extent of eight ounces since last report, (28th October,) and the incision to-day appears healed. He has much cough, and has expectorated since last night, three times the fill of the spit-box, (holding about twenty ounces.) Complains much of pain about the nipple." It appeared to me that the external air, which had got admission, was keeping up the irritation, and that, as it naturally rose to the upper part of the cavity, and the tendency of the wound to heal up with probably the formation of false membranes, prevented its escaping in the same way as it entered, it would be advisable to make another opening higher up, where the blowing out of integuments intimated a communication already existing betwixt the pleura and integuments. Pent-up air is injurious, but air applied to an open surface, no matter of what extent, is not nearly so. Accordingly, to allow of a free exit to the air, an opening was made in the centre of the emphysematous swelling, and the lower wound was again opened. Both openings were after this time kept open with a sponge tent, and the openings were, by this means, enlarged to the full space betwixt the ribs, so that by placing a *speculum vaginæ* over either of them, and holding a candle to the end of it, the *pleura pulmonalis* could be seen of a vivid red colour, and its distance from the ribs ascertained. Twelve ounces of fetid pus came from the upper opening, and next day twenty-four ounces. On the day following, there was the same quantity. After this, the case went on for some time very well, the pus not being above four ounces daily, often much less. On the 16th November, the report says, "Yesterday about three ounces of pus; after which two ounces of a solution of sulphate of zinc, in the proportion of one grain to the ounce of water, were thrown into the upper opening, and allowed to remain for a few minutes. It gave very little pain, and run out on his turning to his belly." On the 20th, "a solution of sulphate of zinc, in the proportion of three grains to the ounce of water, has been injected. Discharge, as before, but thinner. It is emptied out daily when he sits up or turns over to his belly." On the 14th, it is stated, that "he has daily a discharge betwixt two and three ounces in quantity, sometimes thick, at other times thin, and rather fetid. The space betwixt the ribs and *pleura pulmonalis*, which, previous to the last injection, was three-quarters of an inch wide, is now considerably less."

Hitherto he had had frequent perspirations, especially during the night, and his pulse was quick. After this time, however, the general symptoms improved very fast,—the heart had passed over to its natural position nearly, and respiration was heard by the stethoscope nearly as well in the left as the right lung. The report on the 27th is, "General appearance considerably improved. Is out of bed daily for a short time. On taking the plug out of the wound at the visit, there is a discharge of a fluid, at first like water, and afterwards a little purulent, the whole not amounting to two ounces; and this is the whole collected in the course of twenty-four hours."

December 6th. "Was continuing to improve till yesterday morning when he had a rigor.—The discharge amounts to two ounces." He now complained of much pain in his right side about the infra-mammary region. He had great difficulty of breathing, slept little, and perspired most profusely; pulse 130. Leeches were applied to the right side, followed by mustard poultices; but the symptoms did not yield, and he died on the 8th of December.

It was ascertained, that, not satisfied with being out of bed, and walking through the ward,—from being most anxious to get home, and believing himself almost quite well, he had gone out, to try his strength, as he said, on the day previous to that on which he had the rigor.

*Sectio Cadaverous*, twenty-six hours after death. Body considerably emaciated; chest gave a clear sound on both sides, and not much more so on the left than on the right side. A dark-colored fluid ran from the upper opening, on turning the body to the side. On raising the sternum, the pericardium was seen nearly in its natural situation, being rather more under the sternum and to the right side than usual, but not to such a degree as to call for particular notice. The upper opening, which was betwixt the fourth and fifth ribs, led into a cavity, the boundaries of which were the diaphragm below, the pleura and lung to the inside, and the walls of the chest to the outside, the root of the lung behind, and above false membrane, which fastened the apex and part of the lobe of the lungs to the ribs. This space held rather more than the open hand betwixt the lung and the wall of the chest. The lower opening betwixt the sixth and seventh ribs was closed by false membranes. There were no tubercles in the lung, which seemed to be nearly of its normal size. On placing it in water, it floated. There was a small piece of the size of a nut, of a white solid cheesy matter, near its root. Around this, the lung was condensed, and showed red hepatization, but not extensively so. The root of the lung was broken in some parts; but this was believed to be owing to its being torn in separating it from the adjacent adhesions, which were very strong. The part of the lung opposite to the upper opening was coverred with a thick layer of a reddish lymph, or rather organized membrane, for the extent of a hand-breadth. The right lung was quite healthy internally, but its lower lobe was covered with large quantities of recently effused lymph,—the result of the pleuritic attack which carried him off.

The *post mortem* examination, it must be observed, was made under adverse circumstances; and the chest was not allowed to be opened in the way that would have been preferred, as that most likely to exhibit the exact state of the part. There were discharged 346 ounces of purulent matter, besides what run into the bed, and could not be collected.

Notwithstanding that the rule in practice to make the opening, where the feeling of fluctuation or the protrusion of the integuments indicates that ulcerative inflammation would probably in process of time achieve this end, is upon the whole most advisable; yet, if this afterwards prove insufficient for emptying thoroughly the collection of fluid, there can be no reason why the same rule should not also apply there as in other cases of abscess, and a more dependent opening be made. I soon became aware that this opening was too far forward, and that the pus secreted from the walls of the cavity did not get a free escape, as shown by its running out latterly only when he turned himself on his belly. Should a case of the kind occur to me again, I would, though performing the operation of necessity at first, keep in view that of choice afterwards; and I intended in this case, before allowing him to leave, and after giving him a little time to ascertain if the discharge would dry up otherwise, to have passed a long probe to the lower and posterior part of this cavity, and made an incision upon it.

It is, I think, almost impossible to prevent the entrance of air even with every caution. The action of the lungs favors the entrance of air in a remarkable degree, and though in many cases the lung of the affected side is not acting, yet the increased expansion of that of the opposite side pushes over the mediastinum, and to a certain extent there is the same expansion and contraction of the area of the affected as of the sound side. False membranes are formed, or adhesions take place, with extreme rapidity in serous membranes; and we cannot rely upon the passage to our external opening being free. It is of the greatest importance to prevent the confinement of air, and the opening for that purpose in the above case was followed by immediate improvement in the symptoms.

There is another circumstance worth notice in the case, relatively to a valuable communication on the subject of empyema in the 17th number of the Dublin Journal of Medical Science, by Dr. Greene, and that is the occurrence of a most abundant expectoration on the temporary closing up of the external wound. The report does not state whether the sputa were fetid, mucous, or purulent; but we gather that the symptoms were not improved. There was no fistulous communication by the bronchial tubes with the purulent collection; and if, as Dr. Greene thinks, there does, in some of these cases, a metatasis take place, or a critical evacuation from the bronchial tubes, there was no relief afforded here. No notice is taken of expectoration after the second opening had been made, so that it had most likely ceased.

Case II.—The following case is against the opinion of Begin, (Dict. de Med. et Chirurg art. Empyème,) as quoted by Dr. Greene, "that of the two methods of evacuation of the matter of empyema, that by thoracic abcess is always more advantageous than the route which the collection sometimes takes by perforation through the bronchial tubes, inasmuch as, in the latter instance, lesions of the pulmonary tissue are often superadded to the primary affection."

Mrs. Bate, aged 40, married, was admitted, 10th December, with typhous fever, accompanied with the usual exanthema of a bright florid hue. On the 15th she had symptoms of pneumonia, attended with cough and rusty sputa. On the 31st, erysipelas attacked a swelled gland which she had in the axilla and extended down the arm, and travelled in an erythematic form over the whole back down to the nates and perineum; and afterwards attacked the face and head. On the 6th of January, the erysipelatous redness was nearly gone, she was sleeping well, her skin moist, and pulse at 80, the face being still swelled and eyelids closed. On the 11th a large abcess was forming under the scapula of the right side, and appearing below its inferior angle. A few days afterwards this abcess was opened. On the 4th of February, the abscess still discharging by the opening, she complained of pain in the axilla, and also of some pain in a soft puffy swelling, but without redness, immediately above the right mamma. She had a painful short cough, without expectoration, and a catch in her breathing for a fortnight past. The pulse was about 100. The whole of this side of the chest emitted a dull sound, and respiration was indistinct, except at the spine and in the infra-mammary region. Can only lie on the side opposite to that where the swelling is. At 3 o'clock on the morning of the 5th of February, she began to expectorate, by an incessant short cough, a quantity of purulent matter, which more than filled a wash-hand basin. The cough and expectoration in the forenoon of that day were not so troublesome, the swelling was much gone, and a gurgling sound was heard when the hand was laid on it. Expiration and inspiration were very loud to the ear applied over the part by the intervention of the stethoscope. Fluid could be pressed from below the external parts into the cavity of the chest, and the point of the finger could close a small opening felt betwixt two of the ribs, thereby preventing the blowing out of the external integuments in respiration. Much heat of skin, thirst, pulse 100. The sputa without odour, sometimes tinged with blood, or of a reddish or pinkish color. On the 8th, the expectoration was much less, but gurgling sound still perceptible. On the 12th, the cough and expectoration had ceased, and she felt easy. No swelling on the side, but a rushing sound still heard by means of the stethoscope. Pulse 72; appetite good. On the 18th February she was discharged perfectly well.

This woman had lost one of her family by phthisis a little before she entered the hospital; and she was known to be strongly predisposed to that disease, and to have had premonitory symptoms of it previous to her attack of fever. It did not appear to me, however, that the collection of fluid in the pleura had any connection with that disease. It has been no unusual circumstance, during the present epidemic, for attacks of erysipelas to take place in the fever patients in the hospital; and a very frequent *sequela* of that disease has been large collections of purulent matter in some part of the body, generally in some superficial part, or numerous small abscesses. Such was the case with the abscess in the back in Bate; and the collection in the pleural sac I regarded as of the same nature, as rather a deposit, than the result of severe inflammatory action, for such never did exist; the deposit being directed to this part, no doubt, by the previous pneumonia or pluero-pneumonia. In the *post mortem* examination of several cases of fever, we found an extensive collection of pus in the pleura of one side; and in many of these the common symptoms had not previously led us to suspect any inflammatory disease in the chest. The continuance or occurrence of dry tongue, heat of skin, wont of appetite, and quick pulse, after the external symptoms of erysipelas had gone off, led us to suspect some collection of pus without local pain, and we were seldom wrong. Where no abscess or internal collection of pus could be discovered, we generally found the urine containing a quantity of pus; and these cases were often very tedious in recovery. Mrs. Bate has had occasion to be several times at the hospital on account of her family. She is quite well; has no cough; and in the chest nothing abnormal can be discovered by percussion or the stethoscope.

*Epilepsy and Apoplexy.*—The following case is in no respect very remarkable, excepting that the *post mortem* examination explained most satisfactorily the cause of epilepsy, and in what way the case had proved fatal by the symptoms of apoplexy.

John Ireland, aged 52, a pensioner, of sober and industrious habits, had been subject for above seven years to epileptic fits at uncertain intervals. He had no fixed pain or uneasiness in his head; and his mental faculties were perfectly sound. On Tuesday the 12th August, whilst proceeding to put on his coat in the workshop, before going to dinner, he fell down in a fit. As this was not unusual, he was not interfered with; but when he had lain insensible for a much longer time than customary, assistance was sent for, and he was found in a comatose state with dilated pupils, stertorous breathing, and occasional spasmodic motions, until seven in the evening, when, after a convulsion, he died.

Not being in town I did not see him alive, but, having frequently seen him before, I was anxious to examine the head, which was done thirty-six hours after death.

Before dividing the scalp, a contusion was observed on the right temple, extending to the eye of the same side, probably, it was supposed, from his striking some bench or stool, or such object in the workshop on his falling.—On removing the skull-cap, the *dura mater* over the middle lobe of the right side, and partly also over that of the posterior lobe, was of a dark blue color, and the finger at once discovered fluid under it. On cutting it open, it was found that there were from two to three ounces of blood, mostly coagulated, lying betwixt it and the brain, and extending downwards into the middle depression in the base of the skull. The anterior part of the middle lobe, where it rests in the hollow of the bone, was rather softened and covered with much blood, and a rent or fissure was discovered extending from the squamous plate of the temporal bone across in an oblique direction the sphenoid bone.—The sphenoid bone from below the transverse process to the spinous process was rough, with several elevated specula, some of which were as sharp as needles. From the quantity and situation of the blood it appeared as if the spinous artery had been the seat of the rupture.

On the anterior and upper part of the left hemisphere, about two inches above the orbit, the finger when drawn across the *dura mater* detected a hard flat substance under it; and it was discovered that there was at this part on its under surface a plate of bone of about one inch long and half an inch broad, smooth on its upper surface where it adhered to the internal part of the membrane, but irregular on its under surface, which was imbedded in the cerebral substance, and part of the latter, in rather a softened state, adhered so firmly to it that the bone could not be removed without bringing part of the brain with it. The arachnoid and *pia mater* adjacent to this part were thickened. No other disease could be discovered in the head.—*Edinburg Med. and Surg. Jour.*

---

*Clinical Lecture on Fissure of the Anus.* By M. Velpeau.—The patient who now occupies bed No. —- is affected with fissure of the anus, a disease which still requires for its elucidation careful research. Notwithstanding the labors of Boyer, Beclard, Dupuytren, and a few other surgeons, I believe that we have much to learn of the causes, symptoms, and treatment of this painful affection; I shall, therefore, embrace the present opportunity of directing your attention to this subject.

Fissure of the anus consists in the existence of a small narrow ulceration seated in the radiating folds at the margin of the anus, and usually attended with very excruciating pain, whenever the patient goes to stool. Before Boyer's time this disease was almost entirely unknown, and if we examine what has been said concerning it by writers who preceded him, we shall be convinced that our knowledge of its causes, symptoms, &c., dates within the last twenty-five or thirty years.

Let us first turn our attention to the *causes* of fissure of the anus. This disease may be excited by constipation, piles, the evacuation of hard fæcal matter, in large masses, by mechanical injury, from the end of a lavement apparatus, for example, &c.; but in many cases it is developed without our being able to discover or trace any probable cause. It arises, in most instances, in a very gradual manner, and soon assumes the characters of other ulcerations about the part; hence we find great difficulty in assigning to it its true and efficient cause; nor do I think that we could produce the disease by artificial means. It exists in individuals of both sexes, but attacks females more frequently than males; it commonly appears between the ages of twenty-five and sixty, but has been observed at the ages of eighteen or twenty. I have seen it in a young man eighteen years of age, and in a girl of twenty-one; children however seem to be exempt from this affection. One of the most remarkable points connected with the history of fissure of the anus is the great pain and suffering by which it is accompanied; effects which we are unable to reconcile with the slight degree of organic injury constituting the complaint. M. Blandin and M. Harvey de Chégoin think that fissures seated above or below the sphincter ani are of an insignificant nature, and heal of their own accord, or under the most simple treatment; while other fissures are attended by the symptoms described by Boyer. This seems to me to be a speculative distinction, for I have seen several cases of fissure of the anus in which violent pain was a prominent symptom, although the affection did not implicate the sphincter muscle of the anus.

One circumstance, gentlemen, connected with the history of this disease, merits particular attention. The sphincter ani muscle is in a state of permanent constriction. But is this a cause or an effect of the disease? Boyer asserted that this constriction formed a principal exciting cause, since division of the sphincter muscle immediately calms all suffering, without any application whatever having been made to the fissure itself. On the other hand, MM. Roche, Sanson, and Blanden contend that the constriction of the sphincter is a result of the complaint, because the ulceration may frequently exist without our being able to discover it. We cannot, it is true, deny that fissure may sometimes exist without constriction, although it be accompanied by the signs of the latter affection, such as violent pain, burning heat, &c., on going to stool. I have witnessed several examples of this: but who can affirm there is no fissure, merely because the surgeon who examines may be unable to find one? The great authority of Boyer is the only cause of our attaching any weight to his assertions, for they have never been confirmed by the result of *post mortem* examinations. Perhaps, however, there may be some means of reconciling opinions on this interesting point in the history of fissure of the anus. Thus, we can understand how a small fissure, being irritated by the passage of stercoraceous matter, may excite spasmodic constriction in the muscular bands underneath it; and again, we can believe that strong spasmodic contraction of the anus, by inducing costiveness, may induce excoriation of the skin about the anus, and thus become a cause of fissure. Under this point of view constriction of the sphincter ani and fissure are two distinct affections which are independent, but have a strong tendency to merge one into the other.

The *symptoms* of the disease are the following: the patient first experiences some pain on going to stool, and for some time after an evacuation from the bowels. The pain gradually increases in intensity and period of duration, and when it has arrived at its maximum, the patient suffers the most excruciating torture.—The sensation excited by the passage of fæces is compared by the patient to the pain occasioned by a red hot iron, or to the tearing asunder the margin of the anus, and brings on a feeling of faintness or threatening of convulsions. In the intervals between each stool, the patient merely feels some lancinating pain or scalding, with a sense of weight about the part, and colic. As the time for evacuating the bowels approaches, the pain is manifestly increased, is most violent during the moment of expelling the fæces, and then gradually declines for a few hours. It occupies a circumscribed space about the margin of the anus, and is often attended by pulsation of the vessels like that which accompanies phlegmonous inflammation. The bowels now become obstinately constipated, and evacuations take place every eight or ten days, unless purgatives or clysters be employed. The patient feels such a dread of going to stool, that he defers the moment as long as possible, although he knows that such conduct will aggravate his sufferings. One patient at the Hôtel Dieu, was heard to exclaim that he would rather die than go to the water-closet, so great was the pain during evacuation of the bowels. Some persons who labor under this disease have recourse to curious methods of avoiding the inconveniences occasioned by the passage of fæcal matter; thus, Boyer mentions the case of a lady who kept a canula constantly fixed in the rectum, to prevent the suffering which she experienced at each evacuation. But fluid stools, or even the passage of air, will occasionally excite very severe pain. Some patients are able to walk about, sit down, or attend their ordinary business during the intervals between the attacks, but others are compelled to keep their beds in spite of the increased heat and pain thus occasioned. The shooting pains extend towards the bladder or uterus, and in some cases to the hypogastric region. The digestion is impaired; the patient eats little, to avoid the necessity of going to stool; he becomes thin and of a yellow hue; the face is expressive of suffering, and on looking at it, one would say that the patient laboured under some severe organic disease; occasionally the slightest movement will excite the accesses of pain; coughing, spitting, blowing the nose, speaking or singing will aggravate it; any excess in eating or drinking will have a similar effect. The presence of the catamenia, also, increases the sufferings of women. The pain is at once excited by introducing any body into the rectum, as the pipe of a syringe, &c., and when we attempt to pass up the finger, we not only occasion severe agony, but feel that it is powerfully grasped by the constrictor muscle of the anus.

On separating the folds of the anus and drawing the rectum gently down, we perceive, at the bottom of some one fold, a small ulcerated fissure about one or two lines broad, by four, eight, ten, or twelve in length. The edges of the fissure are generally free from hardness; they are of a bright red colour, and bear the strongest resemblance to the cracks which so often exist in the hands, feet, or corners of the mouth. A very small quantity of pus may be discharged from the fissure, and in some cases a little blood. It often happens that we find it extremely difficult to discover the fissure, hidden as it is in the folds of the anus, which is usually, in such cases, more or less of a funnel shape; we must carefully unfold the integuments, and desire the patient to make a few expulsive efforts. The fissure is then exposed; it may occupy any point of the margin of the anus, may scarcely reach the edge of the mucous membrane, or may be confined to the parts above the sphincter. In many cases the fissure seems to commence from or terminate in a pile; and usually it extends in a vertical direction upwards. We can often assure ourselves of the existence of this lesion by the mere touch; the instant the finger comes in contact with the fissure, the most violent pain is experienced by the patient, and we feel a hard, wrinkled cord, which indicates the precise situation of the crack. Such, gentlemen, are the ordinary symptoms of fissure of the anus; severe burning at the moment of going to stool; narrow, elongated and superficial ulceration at the edge of the anus; and violent contraction of the sphincter muscle.

*Progress of the disease.*—Fissure of the anus does not present all the symptoms now enumerated, from its commencement; it begins with very slight pain, or itching; tickling feel or creeping sensation, with heat after each stool. These symptoms may continue for six months, or even a year, before they become sufficiently severe to excite much attention. In other cases, the disease will acquire its greatest degree of intensity in a few weeks, or may be very severe from the commencement. You are not, either, to imagine that the series of symptoms already described will present itself in every case; many patients feel no pain whatever; others (and the patient on whom I am about to operate is of this class) suffer severe and almost constant pain. Our patient has a long fissure, with grayish, irregular, and granulated base, resting on indurated cellular tissue. She suffers much from burning pain whenever she goes to stool; the anus contracts violently, and it is with the utmost difficulty that we can introduce a canula, or even the tip of the little finger. You should therefore remember that Boyer's description of fissure of the anus is merely a general one, and that several varieties may present themselves to us, in which the symptoms described by Boyer do not exist, and to which the treatment recommended by him would be inapplicable,

*Treatment.*—Some cases of painful fissure may get well of their own accord. I knew a medical student who laboured under this disease for seven or eight years, and then recovered without operation or any treatment. A few days ago I saw a patient in town who equally got well without treatment, after three or four years. However, patients are in general very anxious to adopt some means of relieving their sufferings, and removing the unpleasant disease under which they labour. This may be effected with or without operation. Several ointments have been employed for this purpose. Boyer obtained a cure in one case by throwing into the rectum two or three spoonfuls per day of an injection composed of hog's lard, walnut juice, sorrel juice, and almond oil, of each four ounces; but here the fissure was attended with slight contraction of the sphincter.

M. Descudé informs us that we can cure the disease with large doses of the oleum hyosciami, and topical applications of mercurial ointment. Some surgeons speak highly of *douches* of cold water, of decoctions of chœrophyllum or poppy heads, &c. In a few cases I have succeeded with white precipitate ointment. Dupuytren employed, with excellent effect, the following ointment, introduced into the anus by means of a tent: extract of belladonna, two drachms; lard, two ounces; honey, two ounces. More recently, some practitioners have spoken in very favourable terms of *monesia*, and one or two cases of cure by this remedy have been cited; but I fear that it will soon meet with the fate of most of our new remedies, which flourish for a time and are heard of no more. When the above mentioned means have been tried (and we must often try them to satisfy our patients) without success, we must have recourse to one of the following, viz., cauterization, dilatation, division of the sphincter muscle, or excision of the fissure. The disease is sometimes cured in the most satisfactory manner by running a stick of nitrate of silver over the whole surface of the fissure. Beclard pretends that he never failed in this way; but other surgeons have not met with the same success; I have tried it myself in many cases without obtaining any benefit, and think that the cases which presented themselves to Beclard, must have been slight, and unaccompanied with contraction of the sphincter muscle; besides, Beclard employed dilatation at the same time. Cauterization can only cure fissure of the anus by modifying the ulcerated surface, and transforming it into a simple wound, which heals like common solutions of continuity. In this way we explain the success obtained by Guy de Chauliac, and Dionis, who cauterized or scarified the ulcers, and by Guerin, &c., who applied the actual cautery, or irritated the surface of the sore with the nail, &c.

*Dilatation*, by the introduction of tents of lint gradually enlarged, into the rectum, has often been attended with the best effects in the hands of Beclard, Dubois, Marjolin, and others. I have employed this mode of treatment with success in some cases, but it is often tedious and painful. In order to shorten the period of pain and diminish its violence, we should employ the largest tents that we can introduce into the rectum. The pain is at first very severe, but as soon as we get to the fourth or fifth tent it is much mitigated; the tents may be covered with any of the ointments which I have already mentioned to you. However, I should remark that the composition of the ointment does not seem to have any effect whatever. I have tried them all, and afterwards common cerate, and found the latter to answer as well. Dilatation then is the chief element of cure in such cases, and I believe that considerable success would attend its use if we could induce our patients to resist the pain which it, in the first instance, always occasions.

*Incision* of the sphincter ani was proposed by Boyer, and recommended by him as the best, indeed the only mode of treating fissure of the anus; his practice has been followed by most of our surgeons up to the present day. Boyer regards this method as infallible, yet several practitioners mention cases in which it has failed. He was naturally led to advocate this mode of practice, because he believed that contraction of the sphincter ani was the chief cause of fissure.

The preparatory steps of this operation are exactly the same as those for fistula in ano. The lower intestines are to be emptied by means of a lavement, or some mild purgative, in order to ensure quietude for some time after the operation. The instruments employed are a straight, probe-pointed bistoury, a common bistoury; a large tent; a T bandage, and all the minor accessories. The patient is placed on the edge of a bed, with the head low, the under limb extended, the upper one flexed, and the buttocks kept widely apart by assistants. The surgeon now introduces the index finger of his left hand into the rectum, guides along it the flat side of his probe-pointed bistoury, and divides the sphincter. Should the fissure occupy the median line in front, he must not cut upwards, for fear of injuring the urethra or vagina. Boyer thought it sufficient to divide the sphincter at any point, without caring where the fissure may be; but I am of opinion that you will do well to pass the blade of the knife through the fissure at the same time that you divide the muscle. When this has been accomplished, you continue the incision upwards and downwards for an inch or two, so as to cut through the whole thickness of the sphincter. A single incision is usually sufficient; but if there be several fissures, or if excessive contraction of the sphincter be present, then we must make a second incision on the opposite side of the anus. When the edges of the fissure are rounded off, hard, and thickened, we seize them with a forceps, and remove the hardened portions either with the knife or scissors.

The dressing is very simple. A tent of lint covered with cerate is placed between the lips of the wound, but its upper extremity must reach about an inch beyond the superior angle of the incision. The space between the buttocks is then filled with lint, and the whole supported with a T bandage. The tent must be supplied every day until cicatrization takes place. I have said nothing of the occurrence of hæmorrhage, for it is almost impossible that any such accident should happen; but were it to arrive, you must have recourse to the usual means of arresting it, with which you are all familiar.

Such, gentlemen, are the methods of treatment generally employed for the cure of fissure of the anus; cauterization, dilatation, incision. The latter treatment is successful in a vast majority of cases; but as some modern practitioners have insisted on the point, that constriction of the anus is the effect, not the cause of fissure, their opinions have produced some new ideas relative to the treatment of this affection. Upon the principles of these gentlemen, I have practised excision of the fissure instead of dividing the sphincter muscle. This operation had been highly spoken of by Mothe and Guérin; I had mentioned it myself in 1832, and some of my operations were published in 1836. The following is the method which I adopted. The patient is placed in the same position as for incision of the sphincter; the point of the border of the anus occupied by the fissure is then seized with a hook, and a couple of strokes of the bistoury on the right and left complete the excision of the fissured part. Sometimes I employed the scissors to remove it, but always avoided cutting the muscular tissue underneath. The operation is soon over, and unattended with pain; I have performed it eight or ten times at least, and have almost always succeeded in curing the patient. In one or two failures I was unable to find out whether the want of success depended on my not having cut out all the diseased structure, or on some other cause. I believe that when the complaint is of long standing, we should divide the sphincter, and at the same time remove the ulceration; that is to say, combine incision and excision together. I shall do this in the case we are about to operate on. The disease here has existed for several years; the ulcer is large, with a grayish, lardaceous base; and it is very probable that simple incision of the muscle would fail to effect a cure. You may ask me, perhaps, why I employ excision, and do not remain content with division of the muscle, a mode of treatment which has been sanctioned by experience. My reasons are the following. Division of the sphincter is an easy and quick operation, and attended almost certainly with success; but it compels us to cut through the deeper-seated tissues beyond the muscle. The wound which results, always suppurates for some time, and may occasion dangerous accidents. The inflammation and formation of matter may extend to the pelvis, and compromise the patient's life. I have seen two cases in which the patients died after a division of the sphincter for fissure of the anus. The operation of incision is entirely free from this danger, because the cellular tissue beyond the sphincter is not touched. The resulting inflammation is very slight, and the wound requires to be dressed for three or four days only. Finally, it is an operation much more simple than division, and one which we should always prefer in recent cases; but when the disease is of long standing, and the contraction of the sphincter violent, we should combine the two operations, so as to ensure success in the most obstinate cases.—*Provincial Med. aud Surg. Journal*, April 3, 1841.

M. Velpeau neglects all mention of the method of treatment proposed by Bretonneau, which consists in the injection of rhatany, and which he found so eminently successful. See Medical Examiner, No. 35, 1840, and No. 19, 1841.

---

*On Resection of the Elbow-Joint.* By M. Roux.—Within the last twenty months I have performed the operation of excision of the elbow-joint three times; one in July, 1839; another in 1840; and the third in the month of September last. These are not the only cases in which I have performed a similar operation; but I submit them to my surgical brethren, chiefly on account of an improvement in the mode of operation, which I believe to be of some importance.

We all know that when white swellings have arrived at a certain degree, the surgeon has no other resource left than to amputate the limb, or excise the diseased joint. Each method of relief has its advantages and disadvantages; but they are not applicable to all joints, nor can they be compared together, except in certain cases. For my own part, I strongly incline to the propriety of excision of some joints, whenever such an operation is practicable. Since the year 1812, I have excised the elbow-joint eleven times, and this sum total exceeds that of all the other important joints put together, for I do not now speak either of the smaller articulations, or of cases in which only a portion of a joint has been removed. Of the eleven operations three terminated fatally, the remaining eight recovered; of some patients I lost sight; but two I had an opportunity of watching for a long time; one of the latter was a chambermaid, and for fifteen years she was able to follow her usual occupations; she is now married, and conducts a commercial establishment; the other, also, was in a condition to follow his trade during ten or twelve years.

In my first eight cases I adopted the common mode of operating. I made two incisions, one on each side of the joint, and a transverse one behind, on a level with the olecranon, so as to expose the hard parts. But this mode gave rise to several inconveniences, particularly with reference to the after treatment and dressing of the wound. Hence I resolved, even at the risk of making the operation more difficult, to modify it, by making an external and a transverse incision only, and thus forming two triangular flaps, united in such a manner that the limb may be retained in a perfect state of rest during the after treatment. I have employed this method in the three cases now submitted to the Academy, and have some reason to believe that it has contributed not a little to their happy termination.—*Provincial Med. and Surg. Journal, from Bull. de l'Acad.*

---

*Wound of the Heart.*—Professor MALLE relates in his *Clinique Chirurgicale de l' Hopital d' Instruction de Strasbourg*, the following remarkable case of wound of the heart.

"A soldier, ætat. 31, was amusing himself with a comrade in firing, when suddenly his friend's gun burst, and wounded him. He fell instantly in syncope, but soon regained his senses and complained of a severe pain behind the sternum. He was carried to the hospital, where he presented the following symptoms: About two inches on the inner side of the left nipple, between the sixth and seventh ribs, there was a small wound, which gave passage neither to blood nor to air. The chest was normally resonant beneath it; the patient had some bloody expectoration; the heart's motions were obscure and the pulse feeble; there was dyspnœa; the skin was cold, the face pale, and the patient felt as if he should faint. The surgeon in attendance, regarding the greatest part of these symptoms as the effect of the fright, ordered some simple means. Four hours after reaction took place, and the patient was bled to ten ounces, after which the expectoration of blood completely ceased. The pulsations of the heart, however, remained obscure, the pulse was small, and the sternal pain continued; but the face had regained its color, the heat of the surface had returned, and the threatenings of fainting had passed away; he was bled again in the evening.

"Next day, the patient had passed a tranquil night; the pulse was fuller and regular, at 100; the face flushed but expressive of suffering; the pain behind the sternum continued; the ear distinguished at the part a kind of undulatory crepitation, something like that heard in a varicose aneurism; and there was a little crepitation in the left lung near the heart. Everywhere else the natural respiratory murmur was heard, though the dyspnœa continued; the horizontal position was irksome. The patient was again bled to ten ounces, and cupped over the heart. On each of the two following days the condition of the patient remained unaltered, and the same depletory means were repeated.

"On the fourth day after the accident he was better; he had slept four hours and had lost his faintness. He had less pain, there was no longer any râle, the pulse was less frequent and regular. The improvement continued during the succeeding days; the patient had some appetite and took some light food. The pain behind the sternum was almost gone; but there was still dulness in the precordial region; the pulse remained feeble, and he did not evidently gain strength. His apparent convalescence, though it advanced slowly, would probably have been more marked had it not been twice or three times interrupted by the patient's heat of temper and refusal of being restrained to perfect quietude. After each fit of anger, his cough became worse, the dullness in the precordial region more extensive, the pain at the sternum more acute, and the pulsations of the heart more obscure; and for every such aggravation of symptoms depletions were required and were generally efficient. He continued thus altetnately improving and suffering from a return or aggravation of his first symptoms up to the forty-second day after the accident, when his condition was such as to permit a hope that his recovery would be permanent. At this time, however, he was seized, without any evident cause, with erysipelas of the left leg, with fever, &c; his old symptoms returned with irrepressible violence, and he died on the 14th of May, having received the wound on the 28th of March.

"On examination the brain and the abdominal organs were found healthy. In the chest at the wounded part there was a cicatrix scarcely firm. The right lung presented, on its anterior surface and near the heart, a small cicatrix, which was also discoverable under the corresponding portion posteriorly, proving that the lung had been perforated. It was also hepatized for about four inches, and united by some slight adhesions to the pleura. The pericardium was larger than usual, and at first appeared distended with liquid. It contained about five ounces of reddish sanies, and some fibrinous clots, two of which adhered to the heart. A foreign body was fixed in the left ventricle. On closer examination this was found to be a portion of the stock of the gun that had burst; it was situated at the front, and about the middle third of the ventricle; its free extremity, which was about as large as a full-sized writing quill, projecting about ten lines; the cavity of the ventricle contained a very firm coagulum extending into the aorta. The piece of wood had traversed the left ventricle and the septum, and projected into the cavity of the right ventricle. Its direction was obliquely from without inwards and from below upwards; its form was somewhar triangular, tapering irregularly from the part that projected in front to that which traversed the septum. The internal surface of the heart was red, and in parts a little softened, especially near the apex; near the valves, on the contrary, the membrane appeared slightly hypertrophied."

*Brit. and For. Med. Rev.*

---

*Reduction of Hemorrhoidal Tumors.*—Much difficulty is sometimes experienced in returning protruded hemorrhoids. Pressure alone is applied in vain. But if the patient be directed, Dr. Marshall Hall says, to make a forcible expulsive effort, and pressure be simultaneously made, the tumor frequently recedes immediately, the sphincter is positively relaxed, the ligature which it formed round the tumor is removed, and the reduction is easy.

Dr. Hall inquires whether a similar measure would aid the accoucheur in returning prolapsus uteri?—*Lancet.*

---

*Influence of Supra-orbital wounds in the production of amaurosis.*—Dr. F. De Walther, after a full and impartial review of the recorded cases of amaurosis, said to be produced in consequence of wounds over the seat of the supra-orbital nerve, arrives at the conclusion, that injury of this nerve has nothing to do with the production of this affection, since the nerve is often divided without giving rise to amaurosis, and amaurosis has been produced when the nerve is left quite entire and unaltered. M. Walther endeavors to prove that the supra-orbital nerve has no direct communication with the optic nerve, or the retina, and that its connection with the ciliary nerves is very indirect, being by means of the nasal nerve. Amaurosis, which occurs after a wound over the supra-orbital region, he therefore attributes either to some direct violence having been sustained by the organs within the orbit, as by concussion, or to an affection of the brain; he however admits that amaurosis may also be produced in consequence of inflammatory action being propagated from the supra-orbital wound, to the retina or optic nerve, through means of the continuity of the tissues.—*Edin Med. and Surg. Journal, from Jour. de Chirurgie, und Augenhielkunde,* October 1840.

